# Supplementary material for: Dysregulation of neuroprotective lipoxin pathway in astrocytes in response to cytokines and ocular hypertension​
Source: Acta Neuropathol Commun. 2024 Apr 12;12:58. doi: 10.1186/s40478-024-01767-2 (PMC11010376; doi:10.1186/s40478-024-01767-2)
Supplement: Supplementary file 2 — Supplementary Material 2 [file 40478_2024_1767_MOESM2_ESM.docx]

**Table 2: Sequence of Human Forward and Reverse Prime**

| **Primers** | **Forward Sequence 5'- 3'** | **Reverse Sequence 5'- 3'** |
| --- | --- | --- |
| *GAPDH* | GGAGCGAGATCCCTCCAAAAT | GGCTGTTGTCATACTTCTCATGG |
| *ALOX5* | TCAACTTCGCCAGTACGAC | TCTGCTCAATGGTCACCACG |
| *ALOX15* | TCAGGTTCCCTTGTTACCGC | GTTTCCCCACCGGTACAACT |
| *FPR2* | AGTCTGCTGGCTACACTGTTC | TGGTAATGTGGCCGTGAAGA |
| *SERPING-1* | GGGATGCTTTGGTAGATTTCTCC | GAGGATGCTCTCCAGGTTTGT |
| *LCN2* | GAAGTGTGACTACTGGATCAGA | ACCACTCGGACGAGGTAACT |
| *C3* | GGGGAGTCCCATGTACTCTATC | GGAAGTCGTGGACAGTAACAG |
